# Intersecting sex and gender diversity with sexual rights for people living with dementia in later life: an example of developing a learning framework for policy and practice

**DOI:** 10.3389/frdem.2024.1349023

**Published:** 2024-01-31

**Authors:** Trish Hafford-Letchfield

**Affiliations:** Department of Social Work and Social Policy, Faculty of Humanities and Social Sciences, University of Strathclyde, Glasgow, United Kingdom

**Keywords:** LGBT+, dementia, intimacy, sexuality, social care, workforce development, learning framework

## Abstract

The proliferation of literature on dementia over the last decade has begun to address the experience of LGBTQ+ people's experiences in later life. Changes in cognitive function can jeopardize the safety, wellbeing, and human rights of LGBTQ+ people if the social care workforce are not prepared or versed in responding to their unique needs. The intersection of age, cognitive function, sexual and gender diversity with the expression of intimacy and sexuality requires sensitive and respectful consideration. Yet, this is currently an under-researched and less understood area in social care practice. This paper highlights the main messages from the different bodies of contributory literature and introduces the national framework in England UK on LGBTQ+ aging and its potential for supporting workforce development to consider its flexibility in supporting services to becoming more nuanced and affirmative in these areas of practice.

## 1 Introduction

There has been a proliferation of the literature on dementia over the last decade that includes the experience of lesbian, gay, bisexual and trans and people with other diverse sexual and gender identities (LGBT+) (Westwood, 2016; Baril and Silverman, 2022; King, 2022; Westwood and Price, 2023). According to McGovern (2014), this clearly situates LGBT+ people's experiences in later life at the ‘intersection of social justice and practice' (p. 845).

Relationships with partners, family, kin, and friends have been shown to be important in their contribution to a person's quality of life, even more so as they get older (Sharifian et al., 2022). For people living with dementia in later life, these play a key role for promoting social inclusion and feeling included in one's own community (Maki et al., 2020). When living with dementia, some will continue to value experiences of love and sexual pleasure either in their existing intimate relationship or in forming new ones (D'Cruz et al., 2020). According to the WHO, regardless of age ‘sexuality is a central aspect of being human and encompasses sex, gender identities and roles, sexual orientation, eroticism, pleasure, intimacy, and reproduction' (World Health Organization, 2006, p5).

A range of research studies have demonstrated that professionals and practitioners in health and social care find it challenging to open up a discussion with older people about sexuality (Simpson et al., 2017; Higgins and Hynes, 2018) where the use of open language and the giving of permission to raise issues can be important (Hinchliff et al., 2023). Professionals talking with service users about sex may perceive this as deeply personal and be afraid of causing feelings of personal discomfort and/or fear that they are transgressing a difficult boundary for the parties involved (Bauer et al., 2016). Further, sexual expression in formal care settings can give rise to ethical concerns ranging from privacy to consent (Chen et al., 2020). These are compounded where there may be cognitive and mental capacity issues and, in some circumstances, where there is sexual and gender diversity. For example, displays of same-sex affection have been shown to jeopardize relationships between LGBT+ individuals with care staff and in friendships with their heterosexual and cisgender peers (Villar et al., 2015; Nowaskie and Sewell, 2021). Where LGBT+ individuals experience cognitive changes, having less control over what they choose to share and disclose to others in care environments and how these might compromise privacy and their sense of agency is concerning, particularly when this involves sensitive information about their identities, their life history and sexual and gender identity (McGovern, 2014). This intersection of age, cognitive function, sexual and gender diversity with the expression of intimacy and sexuality requires sensitive and respectful consideration. However, this is an under-researched and less understood area in social care practice.

This paper gives an overview of what is known about LGBT+ people living with dementia and their expression of intimacy and sexuality. Its focus is on identifying the implications for knowledge, skills, and values in the social care workforce and to consider the need for professional and practice development opportunities and the range of support from the workplace. The role of LGBT+ awareness and targeting LGBT+ issues in professional and practice education and training has been empirically linked to success in providing affirmative care for people in later life. Several studies (Bell et al., 2010; Hughes et al., 2011; Hafford-Letchfield et al., 2023) have identified how the use of policy and benchmarking standards are important to providing a mechanism for linking education to service change and improvements. The author has been involved in two systematic reviews of education on LGBT+ aging which sought to firstly identify the main pedagogic principles that should underpin workforce development (Higgins et al., 2019) and secondly to identify the range of interventions that have been used to with the health and social care workforce and how effective the outcomes have been (Jurček et al., 2021). Recommended areas for improvement from this body of research included giving attention to how and where LGBT+ content is included in the health and social care curriculum and the role of teaching and assessment strategies which support their inclusion, for example, through participation of people with lived experience. These two reviews highlight how important it is for professional education to make their standards, benchmarks and learning outcomes on LGBT+ more explicit and how these intersect with the role of education in addressing inequalities in later life and the broader issues of care that impact on LGBT+ populations (Higgins et al., 2019; Jurček et al., 2021).

An integrative review by Chen et al. (2020) sought to establish the knowledge, attitude and tools used by health care professionals to assess sexuality focusing on LGBTQI people living “with/(out)” dementia (p. 398). Chen et al. (2020) highlighted three themes which described how the knowledge and attitudes of health care professionals were variable but within the studies examined, there was a paucity of detail on the actual areas of knowledge relating to sexuality and aging (p. 408). They also highlighted how the need for provision of professional development opportunities needed direct support within the workplace. Findings from Chen et al.'s (2020) review also specifically commented on the lack of validated tools to assess knowledge and attitudes toward sexuality in older people living with/(out) dementia. Scharaga and Chang (2021) discussed the inherent problematic nature of using neuro-psychology measures to assess cognitive functioning in people who are trans and non-binary. Binary gender as a normative variable was critiqued for not being culturally sensitive or capable of taking account of psychosocial factors in trans aging (Scharaga and Chang, 2021). There are implications for greater diversification of intervention tools and methods to use with LGBT+ people living with dementia in later life. This extends to a greater need to involve people with lived experiences in the design, delivery, and evaluation of educational interventions (Hafford-Letchfield et al., 2023).

This paper attempts to summarize the key messages from the literature across these different domains and having established the evidence for need, introduces a case study of a national educational initiative in England, UK (Skills for Care, 2023). The LGBTQ+ learning framework (Skills for Care, 2023) “aims to provide a foundation for identifying the insights, knowledge, understanding and skills that the social care workforce need to help them work affirmatively, inclusively, and effectively with individuals from gender and sexually diverse communities” (https://www.skillsforcare.org.uk/Support-for-leaders-and-managers/Supporting-a-diverse-workforce/LGBTQ-learning-framework.aspx) in relation to the different challenges and unique situations that they may face. This initiative represents the first step to formally embed LGBTQ+ standards into education for the social care workforce. Firstly, the flexibilities and transformative potential of the framework approach and its content are described. Secondly, the merits and potential for benchmarking workforce development is discussed in relation to how this might enable support to people with complex identities and needs such as those living with dementia in relation to the expression of sex and intimacy. The LGBTQ+ learning framework enables an intersectional approach to understanding how identifying with two or more minoritized population can support people expressing their uniqueness (Leonard and Mann, 2018; King et al., 2019). The framework provides opportunities for social care professionals' and providers to acquire the knowledge and skills to work with the diversity of backgrounds of LGBT+ older people and the cumulative factors that may lead them to engage with services and the experiences that they bring to this (Nowaskie and Sewell, 2021).

## 2 LGBT+ people and dementia

In the UK, there are an estimated 944,000 people living with dementia and the projected costs of caring is said to reach £25bn (Alzheimer's Research UK, 2021). The number of older LGBT+ community members who are and will be impacted by dementia appears to be significant. One estimate LGBT Foundation (n. d.) has asserted that 68,000 LGBTQ+ people are currently living with dementia. More widely, the combination of an aging population and increasing levels of people openly identifying as LGBT+ with greater acceptance over time, is likely to at least double these estimates by 2030 (Caceres et al., 2020).

The lack of systematic collection of routine large-scale quantitative data on the older LGBT+ population in the UK, has contributed to their invisibility in national statistics and epidemiological research (Beach, 2019). This is the case for many global regions (DiLorito et al., 2022). Pervasive attitudes in society have discouraged LGBT+ older people from “coming out” and being counted. Therefore, achieving an accurate profile of the LGBT+ population is problematic and contributes to an overall lack of knowledge about the lives and needs of the LGBT+ people with dementia in later life including the current aging cohort and younger gay(be) boomers (Ramirez-Valles, 2016). Whilst this is changing, the latter generation may be strong advocates for responsive services with higher expectations and low tolerance for gaps. They are likely to have more permissive attitudes toward expression of sex and intimacy. The UK Census (Office for National Statistics, 2020) introduced a question about sexual and gender identity for the first time in 2021, from which 3.2% identified as gay, lesbian, bisexual, or another sexual orientation (LGB+), such as pansexual, asexual, and queer, and 0.5% said their gender identity and sex registered at birth were different including trans, non-binary and other. Prior to this, the UK Government had conducted a survey of the UK LGBT+ population in 2017 and 108,000 individuals responded. Subsequently, the Government Equalities Office (2018a,b) developed an action plan in response to the stark and continuing inequalities that emerged from the survey data analysis. This included the appointment of a National Advisor for LGBT Health and for the National Health Service (NHS) England. This was probably the first time that a formal and more precise picture of LGBT+ people's needs was painted with further insight into their diverse characteristics and how these compared with the wider UK population (Office for National Statistics, 2020). Despite these developments however, to date, there has been little focus on older LGBT+ people within these initiatives and there is a lack of nuance in the profile of those who will require support where they are living with dementia.

As touched upon earlier, for LGBT+ people and their carers', the known challenges can become more complex for those developing cognitive impairment for people and in their interaction with care services. McGovern (2014) asserts how losing both oneself and one's LGBT+ identity contributes to a double jeopardy of invisibility. This intersectionality of sexual and gender identities, disability, and ageism for LGBT+ people living with dementia carries a high risk of greater isolation (King, 2022), not helped by the absence of studies that directly examine dementia prevalence or cognitive functioning in LGBT+ older people (Correro and Nielson, 2020).

There has been some scientific research into specific risks for cognitive decline for LGBT+ people. This has explored elevated levels of stress hormones associated with accelerated brain aging and cognitive decline and linked chronic minority stress with increased risk of dementia (Hatzenbuehler, 2016). Kim et al. (2023) for example identified increased risk for subjective cognitive decline in trans aging experience through the examination of longitudinal data on determinants of physical functioning and health related quality of life among 855 sexual and gender diverse older adults with cognitive impairment. Scharaga and Chang (2021) have noted the paucity of longitudinal studies focusing on the cognitive effects of sex hormones in trans populations albeit, this is a complex and nuanced area to evaluate. Smith et al. (2022) conducted a scoping review of empirical research on the lived experience of LGBT+ people with dementia and their care partners. Just one study from this review included trans people (see Barrett et al., 2015). They noted that none of the included studies had an explicit focus on intersex, non-binary or queer people making it difficult to elicit any nuance and distinctions between the diverse identities and experiences of older LGBTQ+ people living with dementia.

## 3 Challenging heteronormativity and cisnormativity in dementia care

Ethical issues in the provision of care have been of increasing concern within the trans community (Willis et al., 2021). Despite increased self-acceptance and gradual legislative changes that recognize human rights, the cumulation of discriminatory experiences throughout the life course, can lead to negative psychological outcomes in later life such as increased anxiety, depression, substance use, suicide ideation and behavior (Westwood et al., 2020; Kneale et al., 2021). Budge et al. (2013) examined both facilitative and avoidant coping mechanisms that mediate distress and transition status, support, and loss (p. 545). There are significant barriers to seeking help. These include stigma and discrimination from staff and lack of awareness, skills, and knowledge in the workforce which area all associated with avoidance of care, late identification of health issues or premature transfer to institutional care (Willis et al., 2021).

A key theoretical approach for examining LGBT+ aging, dementia and sexuality is through the critique of normative frames about temporality and memory and how this is both shaped by, and shapes social meanings and their social contexts (Gjødsbøl and Svendsen, 2019, p. 47). Kings' queering of dementia (2022) argues against framing dementia in ways that emphasize the decline and loss that are always noted. He asserts that this overlooks the contexts in which dementia is experienced and the social and cultural factors that are shaping these (see also Kitwood, 1988). Through analysis of how dementia is understood culturally in popular media, King (2022) highlights how normative cultural texts in UK society are framing the “multigenerational family” (p. 4) and the concept of being ‘isolated and alone with dementia' (p. 1). This framing of dementia in what King asserts are “heteronormative, reproductive, futurological terms” (p. 1) and their construction in a cisgender and heterosexual context effectively silences or writes LGBT+ people out of the overall picture. King highlights alternative relational possibilities which “pivot around caring, or broader social networks to find queer ways to live in the world, regardless of cognonormativity” (p. 7) and the role of policy makers and practitioners in this challenge.

Alongside the barriers that trans older people face in care, the scarce literature suggests very particular ways in which gender identity is impacted by cognitive changes. Baril and Silverman (2022) developed a first typology of intervention strategies to mobilize their new paradigm for intersectional analysis. Whilst this does not cover sex and intimacy directly, their analysis helps to address crucial questions that arise on how to work with people who may be living in the past, in relation to intimacy in care and the implications for developing safe and affirmative practices that support this. Baril and Silverman (2022) discuss the everyday situations of care for example, the use of pronouns, intimate physical care (such as help with shaving, hairstyles, and clothing), the gendered interactions that take place socially, and the importance of nuanced health care. In their critical reflections on approaches to ‘gender confusion' where re-transition may be possible for trans people living with dementia, they argue for a trans-affirmative, and anti-ageist, crip and age-positive approach that is flexible enough to support the right to gender self-determination in later life. Taking a trans-affirmative fluid approach is important to acknowledge and support the potentially changing nature of gender identity and expression where there are cognitive changes. In practical terms, formal carers should be willing to diversify their responses which may include thinking about how to be more gender-neutral and promote person-centered support and which also recognizes the structural realities that trans people must deal with (Baril and Silverman, 2022). What all these approaches recognize, is the socially constructive nature of cisgender/cisnormative environments which seeks to pathologise gender. In summary, trans people who have fought for recognition, often over a lifetime, have talked about how fearful they are of the lack of control that they may experience as a result of dementia, threatening both their independence, sense of gendered self and forced dependence on care workers for gender affirmative, often, intimate personal care (Page et al., 2016).

In relation to expression of sex and intimacy, the disinhibition often associated with dementia, if handled well can encourage gender exploration without shame and guilt and move away for interpreting potential gender ambivalence as a *symptom* of dementia (Baril and Silverman, 2022). Trans people's sexuality and intimacy has tended to be viewed as artificial or less real where a preoccupation with their gender or ‘transition' can render them sexless or fetishised (Tomkins, 2014). Transition in later life involves bio-psycho-social impacts and for many will be a positive route to happiness and congruency (Scarrone-Bonhomme, 2021). Trans peoples experiences of negotiating intimate relationships are often overlooked and their histories and experiences of dysphoria may impact on their capacity or even willingness to enter intimate relationships at points in their lifecourse. They may have taken a more pragmatic approach for example, in their existing or new relationships, by adopting celibacy or asexuality (Riggs et al., 2018). For others, the pursuit for authenticity and becoming oneself in later life, may represent a second adolescence and age of rediscovery involving sexual experimentation (Scarrone-Bonhomme, 2021). In an empirical study of trans intimacies Riggs et al. (2018) call for practitioners to be mindful in the way in which they engage with trans people's experiences of intimacy to ensure that a positive focus is incorporated within assessment on what can be fulfilling and meaningful to individuals and how they determine this. Trans people who transitioned earlier in their life may bear the physical signs and scars that ‘out' their history as a trans person and compromise their privacy and dignity during intimate encounters (Siverskog, 2014). Witten's (2016) research found that a small fraction of trans-lesbian identified people considered ending their life prematurely rather than face the trauma of abuse that may come with having dementia or other forced intimacies with care in later life. These findings again highlight the need for flexible support and challenges to what our normative understandings are of what constitutes “appropriate” expressions of sexuality in later life (see also Chasin, 2015).

## 4 Caring, partners, sex, and intimacy

LGBT+ care givers add another dimension to the review of LGBT+ people living with dementia in relation to expression of sex and intimacy. LGBT+ carers tend to be invisible, and the evidence reveals that compared to their heterosexual and cisgender peers, that they experience more loneliness, poorer health, and strain on their resources, particularly financial. These distinct challenges can impact on their care which is often needed or preferred in relation to what is available (DiLorito et al., 2022). LGBT+ carers are often living without children and are younger (Addis et al., 2009). Support services for informal caregivers are unsuitable (Kittle et al., 2022). When one partner has dementia, the progressive loss of individual and shared memories, which provide meaning and support and the loss of these narratives important for partners' shared identities, can be very distressing (McGovern, 2014). Partners have been known to pass themselves off as a friend or relative where this is easier rather than having to explain their relationship to professionals or providers who have not shown openness to this.

There is increasing evidence on the physical and emotional benefits of sexual expression in care settings. For example, Redelman (2008) documents the biomedical effects of releasing neurotransmitters through masturbation and physical touch which in turn promote warmth, muscle relaxation, pain relief and improved quality of sleep. Within palliative care, there are psychosocial benefits of strengthening relationships at the end of life through sexual expression and physical intimacy. The development of practice guidance with older people (BASW., 2018; Hafford-Letchfield, 2021) has encouraged social care to being more active in facilitating people's expression of their sexual and gender identities, personal autonomy, and equality in relationships. This is supported by guidance in the UK Government (2014) which articulates sexuality in its wellbeing principles and checklist. Bradford et al. (2013) offers the following quote which illustrates the challenges in this area, however:

“*There was one lady who did not have a partner, but we could tell she preferred women to men. Sometimes the care staff did find her trying to touch other women, which for some reason either provoked complete outrage or extreme amusement amongst the staff. Neither response was appropriate and it just served to remind me how much work we still needed to do on this issue*” (Bradford et al., 2013, p. 10).

Traies (2016) study of four hundred older lesbians showed that lesbians remained sexually interested and active into later life and articulated a diversity of women's erotic experiences. Their definitions of intimacy included emotional, physical, *and* sexual aspects. Simpson (2012) demonstrates how gay men's friendship networks have been shown to help develop resources such as political and emotional, to negotiate and challenge the stigma of gay aging and to create what he calls ‘friendship families' (p. 82) characterized by ethics of care and mutual understanding within these relationships. However, older gay men are not a homogenous group and will have had different experiences of forming intimate relationships. Practitioners are required to recognize and avoid subconscious judgements concerning gay and bisexual men's involvement in non-monogamous relationships. It means not always assuming that sexual health is a key concern in their relationships, and instead focusing on other aspects of their relationships and helping them to stay connected if they are impacted by dementia and other health issues. This may include online contact particularly for those living in rural areas (Fenge and Jones, 2012; Simpson et al., 2018). Whilst not addressing cognitive issues directly, Fish (2019) research on gay and bisexual men whose sexual lives were impacted by prostate cancer, highlighted some of the nuances of how services were not oriented to gay and bisexual men's needs to adapt and maintain their sexual practices. She suggests the importance of providing specialist advice including peer support groups. For bisexual older people (Almack et al., 2018) life course experiences in their relationship histories may lead to what they call ‘institutional harms' (p. 142) which may be individual, organizational, or structural. When working with bisexual people who are in single, monogamous same-sex or different-sex couple and polyamorous relationships it is easy to make assumptions about the significant people in their life (Almack et al., 2018).

## 5 Improving capacity for cultural sensitivities

Whilst there is minimal literature that addresses the intersection between sexual and gender diversity, age and cognitive status and expression of sex and intimacy, looking across the themes emerging from the literature above, there are some clear messages about the potential role that professionals and practitioners play in normalizing or usualising sex and intimacy in their day-to-day contact with older adults regardless of care contexts. As the earlier example demonstrated, determining whether (and which) sexual acts are tolerated, infantilised, or defined as a behavioral problem (Mahieu et al., 2016) is perceived as an expression of power and leads to discrimination (Hafford-Letchfield et al., 2018). Environmental constraints, routine and lack of privacy or trust may hinder opportunities to negotiate spaces for intimate relationships or lend toward surveillance (Hafford-Letchfield, 2021). Education and awareness that increases the understanding that sexual expression may be a straightforward expression of sexual needs, a need for closeness and comfort, indicate boredom physical restlessness, or result from unmet care needs, can all help to address uninhibited sexual behavior or hypersexuality sometimes noted (International Longevity Centre (ILC), 2011). The guidance accompanying the UK Mental Capacity Act (Department of Health, 2005) asserts the principles of informed consent and decision making and indicating where expert assessment is required. A history of an LGBTQ+ individuals past sexual practices and how they communicate their consent will need good communication skills so that the professional is both willing to discuss the situation, remain non-judgemental and affirmative. This may involve other professionals on a need-to-know basis and how to provide opportunities for people to continue their sexual relationships in a way that affords privacy, dignity, and respect.

## 6 Developing the workforce: the LGBTQ+ learning framework

As stated earlier, sensitivity, familiarity, awareness, capability, and compassion are all essential to be able to demonstrate the skills, knowledge, and values for working with LGBT+ people in later life. They are embedded in the principles of person-centered care and care professionals; practitioners and providers should also engage with the individuals history and current needs as well as promoting human and legal rights. The role of education and training has been well documented (Higgins et al., 2019; Jurček et al., 2021; Hafford-Letchfield et al., 2023) and warrant positive action. Hafford-Letchfield et al. (2023) noted that educators, staff, and activists who identify as LGBT+ are often depended upon to take up leadership roles in development of affirmative LGBT+ care rather than this being enshrined and embedded in other equalities and mainstream education initiatives. While we have a range of legislation, guidance, and research resources, until recently there has been no coherent framework for auditing the outcomes or context in which these are implemented (Hafford-Letchfield et al., 2018, 2023). Education and training alone will not enable or resolve the more systemic organizational change needed for addressing discrimination, and exclusion and the lag of LGBT+ inequalities within wider equality, diversity, and inclusion work (Higgins et al., 2019).

The remainder of this paper introduces the UK Skills for Care LGBTQ+ Learning Framework firstly, by describing the flexibilities and transformative potential of a national framework approach and secondly the significance of benchmarking for workforce development. The framework supports an intersectional model for responding to multiple and complex identities in LGBTQ+ aging that tackles in a nuanced way the expression of identities for LGBTQ+ people with dementia, sexuality and dementia (Leonard and Mann, 2018; King et al., 2019).

The LGBTQ+ Learning Framework aims to set out the core knowledge, skills, and values required by the social care workforce for “working affirmatively, inclusively, and effectively with LGBTQ+ people in later life” and it was launched by Skills for Care, England in 2023 (Skills for Care, 2023). Skills for Care is a national organization funded by the UK central government to provide strategic workforce development and planning in adult social care in England (http://www.skillsforcare.org.uk). It works with social care employers, Government, and a range of strategic partners and they commissioned the framework within their equality, diversity, and inclusion strategy. The Learning Framework was developed by the author in partnership with a national LGBT advocacy organization and a small group of older LGBT+ people with lived experience. The process of developing the framework was therefore participative and drew on co-production in the way it engaged people with lived experience alongside organizations, researchers, and practitioners, all with expertise and/or practice experience in LGBTQ+ aging. The foundation of the framework was informed by a rapid scoping review of the cumulative research evidence which documented the concerns and challenges for sexual and gender diverse communities in their experiences of accessing and using care services. Care was taken to document the strengths, contributions and recommendations made by these stakeholders (including allies and advocates) for how the workforce can work toward operational and system improvement (Skills for Care, 2023). Based on these foundations, the learning framework embodies a structure and content which takes a systemic approach to articulate all the issues and areas that are unique to LGBTQ+ aging. These emerged from the research and were synthesized in the form of 19 key topic areas. Each topic was designed to stand alone as well as being themed and mapped to a domain of care practice that again could provide a modular approach to learning (see Table 1 below for a list of topics and domains). Whilst the framework did not aim to be a complete guide to LGBTQ+ aging and affirmative care, it addresses a very wide audience including frontline learners, practitioners, and providers, those responsible for workforce development such as trainers and professional educators and those in management and leadership positions or responsible for commissioning and contracting and other quality assurance roles. Each topic also maps out the essential knowledge, skills and values that help to underpin and enable better engagement with the delivery of improvements for people in later life. The framework can be developed and expanded to support changes in social care and complements other educational and learning frameworks as well as providing an ongoing conversation for continuous improvement to embed and expand practice.

**Table 1:** Mapping the domains and subjects to the tiers of the workforce in the learning framework.

**Domains and subjects**	**Tier 1**	**Tier 2**	**Tier 3**
**DOMAIN A: LGBTQ**+ **awareness and affirming practice**			
AD1: LGBTQ+ awareness, history and culture	√	√	√
AS2: Language, terminology and communication	√	√	√
AS3: Legal framework, human rights and ethics	√	√	√
**DOMAIN B: health and wellbeing in later life**			
BS4: LGBTQ+ inequalities in later life		√	√
BS5: Family, kinship, communities and networks	√	√	√
BS6: Challenging discrimination, oppression and violence	√	√	√
BS7: Participation, user involvement and co-production	√	√	√
**DOMAIN C: personalized care and support**			
CS8: Intersectionality in LGBTQ+ aging	√	√	√
CS9: Support and care for LGBTQ+ individuals with dementia	√	√	√
CS10: LGBTQ+ carers		√	√
CS11: Sexuality and Intimacy in Later Life		√	√
CS12: Trans affirming care	√	√	√
CS13: HIV and LGBTQ+ aging			√
CS14: Safeguarding LGBTQ+ adults in later life	√	√	√
CS15: End of life care		√	√
**DOMAIN D: leadership, education and service development**			
DS16: Providing inclusive and affirmative care environments		√	√
DS17: Improving services and practice based on research evidence and evaluation		√	√
DS18: Leadership and transforming services for LGBTQ+ individuals and communities			√
DS19: Creating learning environments			√

Reprinted with permission from Skills for Care England.

The process of developing the framework enabled the capturing of a diverse range of freely available e-learning resources which are mapped to the 19 topics to support learning and implementation. Many of these open resources were developed from research findings for the purpose of exchanging knowledge to inform policy and practice. The resources also provide personal narratives of LGBTQ+ people in later life with lived experience as well as their representatives and advocates which places person-centered care at the heart of learning. Other key features of the framework are in how it describes tiers of core knowledge and skills as learning outcomes for three different streams of the social care workforce, for example, (1) those not directly involved in providing care but where their work involves regular contact with people in later life, (2) those working face to face with people in later life and providing assessment and care services and (3) those working in complex areas of decision making and in workforce development who have direct responsibility for developing affirmative services and promoting and disseminating best practices.

The framework articulates how the nineteen subject areas align with four key domains or practice areas of concern to the social care workforce (see Table 1). Each topic comprises a summary of the key messages from the research evidence. The suggested target audience and maps the key learning outcomes for each of the three tiers of the social care workforce to differentiate the level of knowledge and understanding required. Each subject is supported by references to relevant statute and/or national standards and any legislative guidance. There are also further sources of guidance and suggested open learning materials. The aim is for the reader to select relevant subjects which may act as standalone areas, for example, AS2 on terminology and communication can be used to inform awareness training, or this could be combined with another subject such as DS16 on affirmative environments to examine how this awareness informs service developments. Some or all the content of subjects can also be integrated into existing formal learning programmes (e.g., on dementia, LGBTQ+ carers, sexuality, and intimacy) to inform, diversify and enrich learning about those areas in a way that is inclusive of LGBTQ+ experience. Distinguishing between the learning outcomes for different tiers of the workforce also allows the subject content to be adapted to the needs of the learner and their unique role and/or care setting. In short, the learning outcomes for each relevant tier and subject within the framework are intended to provide a clear focus for those involved in training and education as well as service development for any individual or team. The flexibility of this approach recognizes and supports an iterative but cumulative approach that benchmarks care capability and practice. It supports a foundation on equality, diversity, and inclusion as well as building toward a more complex approach and enables organizations to prioritize and plan in relation to future affirmative care. Everyone, for example, would be expected to be familiar and confident on inclusive language but those working with LGBTQ+ people with dementia, involved in assessment and provision would need to know how to actively engage with different identities. Further, those in leadership roles as articulated in Domain D (see Table 1) would be focused on translating the human rights that come with expression of different gender and sexual identities into policies and procedures. These in turn lead to demonstrative improvements in practice and the allocation of appropriate resources and support for the workforce to do so.

In relation to the focus of this paper, there are three topics relevant to supporting LGBTQ+ people living with dementia with their expression of intimacy and sexuality (CS9: Support and care for LGBTQ+ individuals with dementia, CS10: LGBTQ+ carers and, CS11: Sexuality and Intimacy in Later Life) (see Table 1). These subjects lie within the Domain C on Personalized Care and Support and build on the foundations of Domain A and Domain B to support an incremental and tailored approach to learning. Individual learners, educators, trainers, managers, and leaders can utilize these to guide and develop both informal and formal learning. For example, this may enable a better informed and targeted training needs analysis through questions related to the suggested learning outcomes in each area and a review of the suggested learning materials for bespoke training and learning interventions. Therefore, there is potential to establish a minimum standard of performance and capability in relation to addressing gender and sexual diversity, dementia and expression of intimacy and sexuality into the assessment and provision of care to older people. Alongside training, organizations can revisit their induction programmes, and look at the knowledge, skills, and values in recruitment processes. Where there are reviews and discussions of service users situations, it may be that the guidance and learning resources can enrich the offer of support to this population as well as be used to benchmark the quality of care in the process of appraisal and career progression. Further, the learning outcomes can be used to demonstrate evidence against any internal or external audits, service standards through record keeping of activities completed.

Through this process, the LGBTQ+ Learning Framework provides the potential to navigate and articulate which skills, knowledge and behaviors support affirmative and inclusive provision to LGBTQ+ people in later life particularly for those who are living with dementia in relation to their expression of intimacy and sexuality. Considering the evidence discussed earlier about the key issues and contexts, the framework provides a strategic resource that can be operationalised to benchmark current skills and knowledge with what is minimally required. At the informal level, individuals and teams can use the framework to recognize and evaluate their own transferable skills, identify, and work out learning activities to meet any gaps. Within the Skills for Care wider strategy, a significant step has been taken to demonstrate how the continuing professional development and career progression of the social care workforce needs to engage more proactively with these significant areas. At the time of writing Skills for Care have also commissioned an evaluation of how the workforce have utilized and engaged with the framework. A key focus will be in how far social care organizations have facilitated partnerships with LGBTQ+ communities to engage with their lived experience and expertise and their plans for demonstrating improved outcomes in its first year.

## 7 Conclusion

This paper has drawn on a range of literature to highlight how changes in cognitive function can jeopardize the safety, wellbeing, and human rights of LGBT+ people in later life if the social care workforce is not prepared or versed in responding to their unique needs. This intersection of age, cognitive function, sexual and gender diversity with the expression of intimacy and sexuality requires sensitive and respectful consideration. Yet, this is currently an under-researched and less understood area in social care practice. Whilst in its rudimentary stages, the case study on the introduction of a national framework in England UK has illustrated the potential for addressing these issues more systematically through workforce development.

At the time of writing, it is early days in relation to how the social care workforce in England will engage with the LGBTQ+ framework and how any evidence can be used to foster improvements in care services and move toward transformation. Hafford-Letchfield et al. (2018) have noted that changes in organization in relation to equality, diversity and inclusion are usually “more evolutionary as opposed to revolutionary” (p. 313) and as we have witnessed from the literature, the wheels of change for LGBTQ+ affirmative care has tended to turn slowly and in the context of gradual social, political, and legislative change. Similarly, those essential changes to meet new legal requirements are not always supported with consultation and the significant resources needed to foster engagement which can undermine motivation and leadership (Mitchell, 2013). The role of learning, both formal and informal can be aligned with equality, diversity and inclusion strategies that supports change in professional practice and incremental service development as the topics in the framework have endeavored to foster. Within the areas of gender and sexuality identity, intimacy and sexuality and dementia care, there is a need to be able to work flexibly and inclusively to ensure that the wellbeing of LGBTQ+ people in later life and their significant others are included in new service developments and that their needs are authentically and empathically identified (Hafford-Letchfield et al., 2018). Further, to complement the introduction of a national framework while well intended and providing structure and content, more research and evaluation can be utilized to demonstrate the softer developments within social care in terms of culture and distributed, participatory and compassionate leadership to address any barriers to learning and to develop a more sustainable culture for change.

## Data availability statement

The original contributions presented in the study are included in the article/supplementary material, further inquiries can be directed to the corresponding author.

## Author contributions

TH-L: Conceptualization, Project administration, Writing – original draft.
